# Supplementary material for: Thromboembolic Events in a Socio-Economically Disadvantaged Population with COVID-19 Admitted to a Medicalized Hotel in Madrid
Source: Int J Environ Res Public Health. 2022 Jun 25;19(13):7816. doi: 10.3390/ijerph19137816 (PMC9265549; doi:10.3390/ijerph19137816)
Supplement: Supplementary file 1 [file ijerph-19-07816-s001.zip › ijerph-1723511-supplementary.pdf]

Supplementary S1. Patients' characteristics according to household income.

	<950 € n=54	Between 950€-1900€ n=65	Between 1900€- 2850 € n=4	> 2850€ n=7
	**% (n)**	**% (n)**	**% (n)**	**% (n)**
**Sex**				
Male	63 (34)	56 (36)	25 (1)	43 (3)
Female	37 (20)	44 (28)	75 (3)	57 (4)
**Age, years**	50.9 ±12.5	57.2±10.3	64 ±13	52.2±17.7
**Country of origin**				
Spain	26 (14)	51 (33)	50 (2)	0 (0)
Other (migrant)	74 (40)	49 (32)	50 (2)	100 (7)
**Socioeconomic characteristics**				
Study level				
Elementary studies	13 (7)	12 (8)	0 (0)	0 (0)
High-school studies	48 (26)	38 (25)	75 (3)	0 (0)
University studies	9 (5)	5 (3)	0 (0)	71 (5)
Missing	30 (16)	45 (29)	25 (1)	29 (2)
Reason for referral to the VCMH				
Living in overcrowding	45 (24)	38 (25)	50 (2)	43 (3)
Cohabitating with individuals with an increased risk for severe illness from COVID-19	9 (5)	9 (6)	0 (0)	28.5 (2)
Homeless, evicted, shelter residents	11 (6)	0 (0)	0 (0)	0 (0)
Undetermined	35 (19)	53 (34)	50 (2)	28.5 (2)
**Length of hospital stay**	6 (IQR 3-9)	5 (IQR 3-7)	6 (IQR 5.5-7.5)	8.5 (IQR 5-12.5)
**Length of VCMH stay**	9 (IQR 5-15)	6 (IQR 6.5-14)	2 (IQR 2-3)	8 (IQR 6.5-9.5)
**Current smoker**	11 (6)	1.2 (8)	25 (1)	29 (2)
**History of alcohol abuse**	4 (2)	3 (2)	25 (1)	14 (1)
**Pre-existing comorbidities**				
Asthma	6 (3)	(8)	0 (0)	0 (0)
Chronic pulmonary obstructive disease	0 (0)	(1)	0 (0)	14 (1)
Obesity	20 (11)	(8)	0 (0)	43 (3)
Dyslipidaemia	26 (14)	(18)	0 (0)	14 (1)
Hypertension	20 (11)	(9)	25 (1)	14 (1)
Heart disease	2 (1)	(4)	25 (1)	0 (0)
Diabetes	9 (5)	(2)	25 (1)	14 (1)
History of thrombotic events	2 (1)	(4)	0 (0)	0 (0)
History of cancer	7 (4)	(2)	0 (0)	0 (0)
Pregnancy	0 (0)	0 (0)	0 (0)	0 (0)
Mental health disease	24 (13)	(13)	0 (0)	14 (1)
**Daily medications**				
Anticoagulant drugs	4 (2)	(1)	0 (0)	0 (0)
Antiaggregant	2 (1)	(6)	0 (0)	14 (1)
Oral contraceptives	2 (1)	(2)	0(0)	0 (0)
Antineoplastics	4 (2)	(1)	0 (0)	0 (0)
